# Optimized Zebrafish In Vitro Maturation with Real-Time Morphometric Workflow Reveals Inhibition by 1,2-Bis(2,4,6-tribromophenoxy)ethane (BTBPE)

**DOI:** 10.3390/toxics14050368

**Published:** 2026-04-25

**Authors:** Tao Xu, Lihua Yang, Yindan Zhang, Huijia Tang, Yue Guo, Yanmin Guo, Mingpu Du, Ruiwen Li, Biran Zhu, Jian Han, Bingsheng Zhou

**Affiliations:** 1 Institute of Hydrobiology, Chinese Academy of Sciences, Wuhan 430072, China; xutao@ihb.ac.cn (T.X.); zhangyindan@ihb.ac.cn (Y.Z.); tanghuijia@ihb.ac.cn (H.T.); guoyue@ihb.ac.cn (Y.G.); hanjian@ihb.ac.cn (J.H.); bszhou@ihb.ac.cn (B.Z.); 2 College of Advanced Agricultural Sciences, University of Chinese Academy of Sciences, Beijing 100049, China; 3 College of Resources & Environment, Huazhong Agricultural University, Wuhan 430070, China; 4 Ecology and Environment Monitoring and Scientific Research Center, Ecology and Environment Administration of Yangtze River Basin, Ministry of Ecology and Environment, Wuhan 430010, China; guoyanmin1027@163.com (Y.G.); dumingpuihb@163.com (M.D.); 5 School of Basic Medical Sciences, Hubei University of Chinese Medicine, Wuhan 430065, China; zhubir@hotmail.com

**Keywords:** oocyte maturation, in vitro maturation, novel brominated flame retardants

## Abstract

Novel brominated flame retardants (NBFRs), including 1,2-bis(2,4,6-tribromophenoxy)ethane (BTBPE), are emerging endocrine-disrupting chemicals, though their direct effects on female gamete maturation remain insufficiently characterized. In this study, we used a refined zebrafish oocyte in vitro maturation (IVM) model integrating germinal vesicle breakdown (GVBD) assessment with real-time, image-based oocyte diameter quantification. The workflow incorporated donor-condition optimization and diameter-based quality control during sorting. Oocytes from donors 4 to 5 months post-fertilization (mpf) showed more consistent diameter dynamics at the dish level than those from donors 3 to 4 mpf. Mixed-sex co-housing was associated with higher GVBD and larger Δdiameter than separated housing, although this comparison should be considered preliminary. Under DHP induction, BTBPE (1–1000 nM) consistently suppressed GVBD and attenuated maturation-associated diameter increases, with a non-monotonic-like response pattern. These findings indicate that BTBPE impairs oocyte maturation competence in vitro and supports real-time morphometric tracking as a practical QC component for zebrafish IVM workflows.

## 1. Introduction

Human reproductive health has drawn increasing attention as a public health concern, with rising rates of chronic endocrine and reproductive disorders [1]. Among female-specific conditions, polycystic ovary syndrome (PCOS) [2], endometriosis [3], and premature ovarian insufficiency (POI) [4] contribute to impaired fertility, while endocrine-disrupting chemicals (EDCs) are recognized as external drivers that can perturb reproductive function [5]. Ovarian folliculogenesis and oocyte maturation are rate-limiting steps in female fertility, and disruption of these processes by EDCs has been associated with reduced oocyte quality and lower fertilization success in clinical populations [6,7]. The consequences extend to wildlife as well. Environmental EDC contamination has been linked to impaired reproduction, skewed sex ratios, and population-level decline in aquatic species, with teleost fish among the most directly exposed [8]. These patterns highlight the need for experimental systems that can support hazard identification for emerging contaminants and clarify how chemical exposures may impair female reproductive competence, with fish models offering a tractable and ecologically relevant platform for this purpose.

Zebrafish (*Danio rerio*) provide a practical and biologically informative vertebrate model for female reproductive research because ovarian development, folliculogenesis, and endocrine regulation are highly tractable in this species, and zebrafish have been increasingly used to investigate ovarian dysfunction and environmentally related reproductive perturbation [9,10]. The zebrafish ovary is asynchronous, containing follicles at multiple developmental stages simultaneously, which makes stage-resolved analysis important for experimental reproducibility [11,12]. Oocyte maturation competence determines fertilization potential. In zebrafish, follicle development proceeds from primary growth through vitellogenesis to final oocyte maturation (FOM), the rate-limiting step for acquisition of fertilizability. The zebrafish oocyte in vitro maturation (IVM) model, therefore, provides a platform for evaluating oocyte maturation and chemical effects at the gamete level while bypassing confounding regulation from the hypothalamic-pituitary-gonadal (HPG) axis. Seki et al. demonstrated that alkaline culture conditions (pH 9.0) with bovine serum albumin (BSA) supplementation maintain oocyte developmental competence in vitro [13], and Zhan et al. refined mechanical dissociation procedures to reduce physical damage during isolation [14]. These advances established the practical foundation of zebrafish IVM.

Zebrafish IVM has since been applied in reproductive toxicology to assess chemical interference with meiotic resumption. Bisphenol A (BPA) inhibits spontaneous zebrafish oocyte maturation by mimicking the nongenomic inhibitory action of estradiol through a membrane-initiated pathway involving Gper and downstream Egfr/Mapk3/1 signaling [15]. In pesticide screening, endosulfan strongly inhibited GVBD, whereas iprodione exhibited progestin-like activity and induced abnormal precocious oocyte aging [16]. Oocytes from zebrafish exposed dietarily to the brominated flame retardant TBCO also showed reduced in vitro maturation, with disruption possibly mediated through MIH-related signaling components such as IGF-3 [17]. These studies support IVM as a useful model for detecting the direct chemical effects on oocyte maturation phenotypes.

1,2-Bis(2,4,6-tribromophenoxy)ethane (BTBPE), a novel brominated flame retardant (NBFR), has been increasingly detected in environmental matrices and biota, with concentrations reaching up to 5.22 ng/L in surface water [18] and 25 ng/L in wastewater treatment plants [19]. Also, BTBPE has been reported in human serum at up to 229 pg/mL (~0.33 nM) in exposed populations [20], indicating measurable internal human exposure. Toxicokinetic studies in zebrafish show that BTBPE distributes to multiple tissues, including the gonads, with partitioning driven by lipid content; this tissue distribution pattern supports biologically relevant ovarian exposure after uptake and provides a pharmacokinetic basis for selecting zebrafish as a model to assess gonadal effects [21]. Sex-specific endocrine responses to BTBPE have also been documented, including preferential alterations in thyroid-related enzymatic activity in female hatchlings of avian embryos [22]. In male zebrafish, BTBPE exposure produced an increasing trend in 17β-estradiol concentrations and the E_2_/11-ketotestosterone ratio, accompanied by upregulation of erα and erβ gene expression in the liver [23]. Subchronic exposure further reduced sperm total motility and progressive motility, increased morphological malformations, and caused histopathological changes in the testes associated with mitochondrial dysfunction [24]. Whether BTBPE directly interferes with female gamete maturation remains insufficiently characterized. Few studies have directly assessed whether BTBPE impairs meiotic resumption or oocyte maturation competence at the gamete level, highlighting a specific mechanistic gap that the zebrafish IVM assay is well-positioned to address.

Reproducibility in zebrafish IVM can still be constrained by practical factors during donor preparation and oocyte selection. Oocyte diameter is widely used for stage-appropriate inclusion, but it is often assessed only post hoc, limiting real-time stage confirmation. To address these sources of variation, we introduced two refinements. First, we optimized donor conditions (female age window and husbandry conditions, including mixed-sex co-housing versus separated housing) to improve oocyte integrity and batch consistency. Second, we implemented a real-time, OpenCV-based diameter-measurement script for stage verification and size-based selection of stage III oocytes during sorting. Using this refined workflow, we assessed the direct effects of BTBPE on oocyte maturation, evaluated primarily by GVBD [25], together with changes in oocyte diameter as a non-invasive morphometric readout.

## 2. Materials and Methods

### 2.1. Chemicals and Reagents

1,2-Bis(2,4,6-tribromophenoxy)ethane (BTBPE, 99% purity) and 17α,20β-dihydroxy-4-pregnen-3-one (DHP) were obtained from Toronto Research Chemicals (Toronto, ON, Canada) and MedChemExpress (Monmouth Junction, NJ, USA), respectively. Leibovitz’s L-15 medium, fetal bovine serum (FBS), and penicillin-streptomycin were obtained from Procell (Wuhan, China), LabRel (Wuhan, China), and Gibco (Grand Island, NY, USA), respectively. BTBPE stock solutions (1 mg/mL) were prepared in dimethyl sulfoxide (DMSO) and stored at 4 °C in the dark. Working solutions were freshly prepared on the day of exposure. DHP stock solution was prepared in ethanol. All reagents were of at least analytical grade.

### 2.2. Zebrafish Husbandry and Donor Optimization

Adult wild-type AB strain zebrafish obtained from the zebrafish breeding center at the Institute of Hydrobiology, Chinese Academy of Sciences (IHB, CAS), were housed in 20 L tanks in a recirculating aquatic system. Environmental conditions were strictly maintained under a 14:10 h light/dark photoperiod and a water temperature of 28 ± 0.5 °C. Fish were fed twice a day with brine shrimp (*Artemia salina*), and water quality was monitored daily. To minimize transfer-induced stress, all fish were acclimated for 14 days prior to experimental procedures.

To optimize donor conditions, females were grouped either by age (3–4 vs. 4–5 months post fertilization) or by husbandry condition (mixed-sex co-housing vs. separated housing) in independent experiments; these factors were not combined in a full-factorial design. The age comparison was conducted under mixed-sex co-housing conditions. The housing comparison was motivated by a practical observation made during the study: when separated housing was trialed during a later phase to expand donor availability, a marked reduction in oocyte performance was observed in those batches, which prompted formal evaluation of housing condition as an independent donor quality variable. For mixed-sex co-housing, tanks were maintained at a 1:1 sex ratio. For separated housing, females were maintained without males under otherwise identical conditions. The care and use of zebrafish was conducted in compliance with the Guidelines of the Care and Use of Laboratory Animals of the Institute of Hydrobiology, Chinese Academy of Sciences (Approval ID: IHB/LL/2024075).

### 2.3. Real-Time Image-Based Oocyte Quantification

Oocyte diameter was quantified using a custom Python-based image analysis script implemented with OpenCV. Live microscopic images were converted to grayscale and processed using the Hough Circle Transform-based circular-object detection. The diameter range used for selection was user-defined. Pixel-to-micrometer conversion was calibrated using a stage micrometer under each magnification setting and recalibrated when imaging conditions changed. To enhance detection reliability, circles were retained only when consistently identified across consecutive frames, and overlapping detections were filtered. Manual correction was performed when necessary. In this study, stage III (late vitellogenic) oocytes were selected using a diameter window of 550–690 μm.

During the development of the Python-based image analysis script, generative AI tools (ChatGPT 5.3-Codex, OpenAI, San Francisco, CA, USA; Claude Opus 4.6, Anthropic, San Francisco, CA, USA; Gemini 3.1 Pro, Google, Mountain View, CA, USA) were used to assist with code drafting, debugging, and optimization of the OpenCV-based circle detection workflow. All AI-generated code was reviewed, tested, and validated by the authors before use.

### 2.4. Oocyte Collection and In Vitro Maturation (IVM)

The complete culture medium consisted of Leibovitz’s L-15 medium supplemented with 10% (*v*/*v*) FBS. The sorting medium was further supplemented with 1% (*v*/*v*) penicillin-streptomycin. The maturation medium was prepared by adding DHP to the complete medium at a final concentration of 1 μg/mL. Adult female zebrafish were anesthetized by ice-water immersion, and ovaries were dissected and transferred into phosphate-buffered saline (PBS) at room temperature to remove residual blood and surface lipids, followed by transfer into sorting medium. Oocytes were gently released by mechanical dissociation using fine dissecting needles. Stage III oocytes were selected using the real-time diameter measurement system described above, and the initial diameter (D_initial_) of each oocyte was recorded prior to exposure. Only oocytes meeting the predefined diameter criteria were used in subsequent assays. Approximately 90 stage III oocytes meeting the diameter criteria were typically obtained from each female during a single dissection.

### 2.5. Exposure Design, GVBD Assessment, and Diameter Change

Selected oocytes were randomly assigned to the following groups:

1. Hormone-free control (without DHP);
2. Positive control (DHP-treated);
3. DHP-induced groups co-exposed to BTBPE at final concentrations of 1, 10, 100, or 1000 nM.

The 1–1000 nM range was selected to characterize the concentration-response profile across three orders of magnitude. The lower bound (1 nM) approximates the upper end of human serum concentrations reported in exposed populations (~0.33 nM) [20], while higher concentrations were informed by a prior in vitro study to support mechanistic interpretation [26]. DMSO was used as the solvent control at a final concentration of 0.1% (*v*/*v*) in all groups. Ethanol content was kept constant across groups. Each culture dish contained 30 oocytes, and all treatments were performed in triplicate. Cultures were incubated at 28 °C in the dark for 260 min [13].

At the end of incubation, the final oocyte diameter (D_final_) was re-measured using the same calibrated image analysis system. For each oocyte, Δdiameter = D_final_ − D_initial_. For each culture dish, the mean Δdiameter was calculated from all oocytes in that dish, and dish means (*n* = 3 dishes per group) were used for statistical comparisons. Oocyte maturation was assessed by determining the germinal vesicle breakdown (GVBD) rate under a stereomicroscope. GVBD, a widely accepted morphological endpoint of meiotic resumption in zebrafish oocytes [27], was defined as the disappearance of the germinal vesicle accompanied by cytoplasmic transparency at the animal pole. The GVBD rate was calculated as the percentage of mature oocytes relative to the total number of oocytes in each dish.

To clarify the experimental sequence: donor age was optimized first under mixed-sex co-housing conditions, after which the housing condition was formally evaluated using 4–5 mpf donors. The BTBPE exposure experiment was conducted using the confirmed optimal conditions from these two sequential experiments (4–5 mpf females, mixed-sex co-housing).

### 2.6. Statistical Analysis

Statistical analyses were performed using SPSS (version 27.0; IBM Corp., Armonk, NY, USA) and GraphPad Prism (version 10.5.0; GraphPad Software, San Diego, CA, USA). Data are presented as mean ± SEM. For GVBD rate and Δdiameter, the culture dish was treated as the experimental unit (*n* = 3 dishes per group). Normality and homogeneity of variance were assessed using the Shapiro–Wilk test and Levene’s test, respectively. For within-group comparisons of pre- versus post-incubation oocyte diameter measured on the same oocytes, a paired two-tailed Student’s *t*-test was used. For comparisons between two independent groups, an unpaired two-tailed Student’s *t*-test was applied. For comparisons among multiple groups, one-way ANOVA was performed, followed by the least significant difference (LSD) post hoc test when ANOVA was significant. Statistical significance was set at *p* < 0.05.

## 3. Results and Discussion

### 3.1. Overview of the Refined IVM Workflow and Donor-Dependent Phenotypes

**Figure 1:**
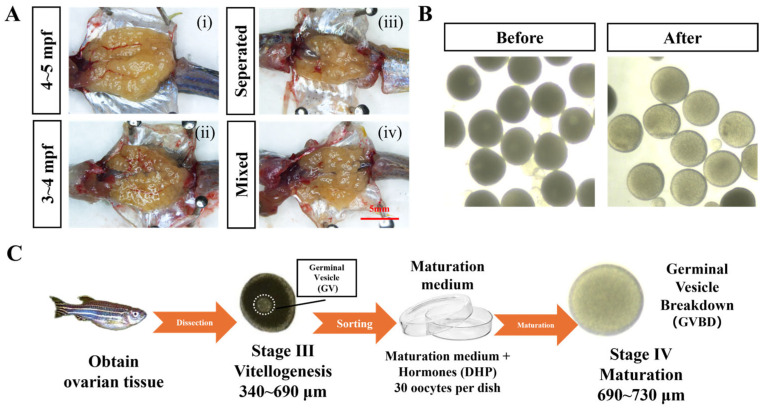
Overview of the refined zebrafish IVM workflow and donor-dependent phenotypes. (**A**) Representative ovarian images from donor groups used in the optimization experiments: (**i**) 4–5 mpf, mixed-sex co-housing; (**ii**) 3–4 mpf, mixed-sex co-housing; (**iii**) 4–5 mpf, separated housing; (**iv**) 4–5 mpf, mixed-sex co-housing. Images are provided for contextual illustration of representative dissection material. Paired images from both housing conditions at 4–5 mpf within the same dissection session were not available due to the sequential experimental design. Scale bar = 5 mm. (**B**) Representative images of oocytes before and after incubation. (**C**) Workflow of IVM.

A schematic overview of the refined zebrafish IVM workflow is shown in Figure 1. The workflow included ovary dissection, mechanical release of oocytes, diameter-based sorting of stage III oocytes, in vitro maturation culture, and endpoint assessment by GVBD, following previously reported zebrafish IVM workflows [14,28], with workflow-level refinements introduced in the present study. Representative ovarian images from each donor group are shown in Figure 1A for contextual reference. Within the 3–5 mpf range examined here, gross ovarian appearance at dissection did not provide a reliable basis for discriminating between donor groups because of individual variation in dissection timing, individual physiological state, and spawning history. This is consistent with the asynchronous nature of the zebrafish ovary [29] and the known dependence of reproductive performance on physiological condition rather than chronological age [30]. Accordingly, donor group selection was based entirely on the quantitative functional outcomes described in Section 3.2 and Section 3.3, not on gross morphological criteria. Formal histological analysis of follicle stage composition across donor groups was outside the scope of this study and will be addressed in future work.

To evaluate the practical efficacy of the OpenCV-based diameter measurement workflow, we summarize the distribution of measured diameters for a representative batch and report the frequency of manual interventions required during detection. Diameter-based staging is widely used in zebrafish IVM to constrain stage heterogeneity. While Stage III is broadly defined as ~340–690 μm, we employed a stricter selection window (550–690 μm) to specifically isolate late vitellogenic oocytes, to reduce stage overlap and improve batch consistency. This distinction is critical to avoid overlapping with Stage IV (690–730 μm) and Stage V (730–750 μm) follicles [31,32] and is generally recommended as a QC variable rather than a standalone maturity endpoint. Manual correction was defined as user intervention to add missed detections or remove false-positive circles during real-time sorting.

The manual correction rate was calculated using the formula:

(1) $$\mathrm{Manual}\;\mathrm{Correction}\;\mathrm{Rate}=\;\frac{N_{\mathrm{manual}}}{N_{\mathrm{auto}}+N_{\mathrm{manual}}}\times 100\%$$

**Figure 2:**
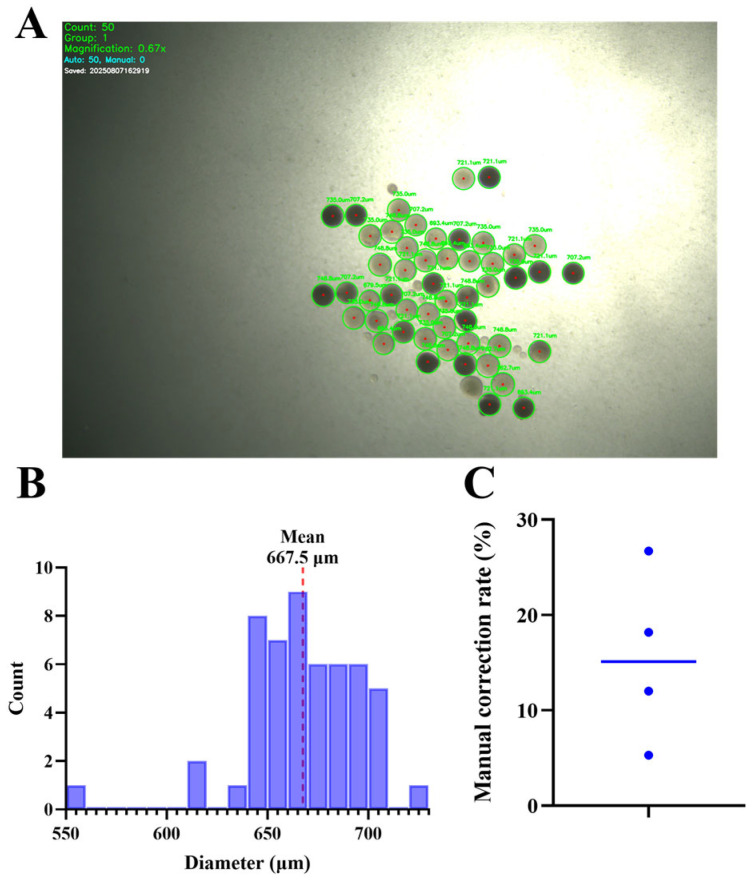
Real-time diameter measurement output and manual correction rate. (**A**) Example of automatic circle detection overlay. (**B**) Diameter histogram for a representative batch. (**C**) Manual correction rate (%) across representative runs.

Across four representative images/runs, manual correction rates ranged from 5.26% to 26.67%, indicating that most oocytes were detected automatically while a minority required user intervention (Figure 2). Together with the diameter histogram and representative detection overlays, these metrics provide an auditable description of how size-based selection was implemented during sorting. In addition, pixel-to-μm calibration was established using a stage micrometer under each magnification setting and applied to the real-time detection workflow.

### 3.2. Donor Age Optimization for IVM: Maturation Outcomes and Diameter Dynamics

**Figure 3:**
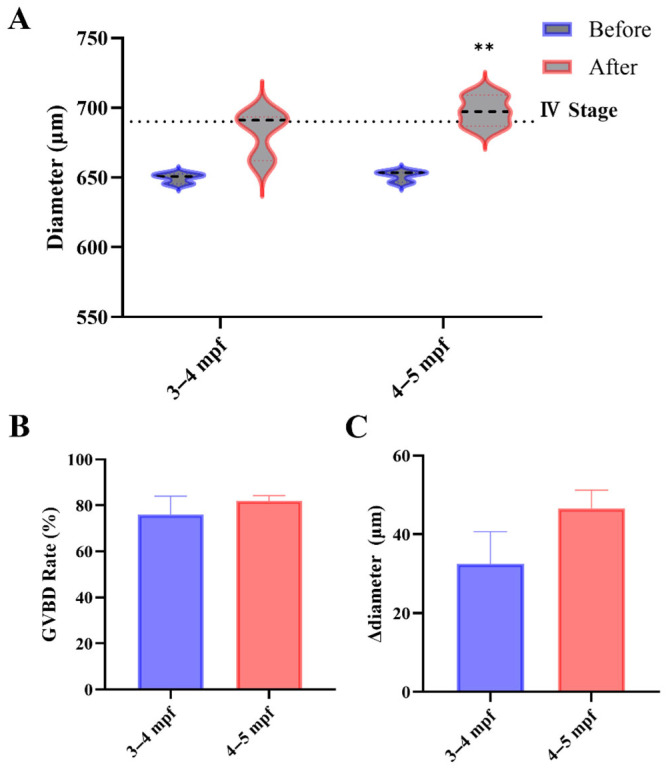
Donor age (3–4 vs. 4–5 mpf) and IVM outcomes. (**A**) Oocyte diameter before and after incubation (paired comparisons within groups). (**B**) GVBD rate. (**C**) Δdiameter (post-pre) summarized as dish means. Data are mean ± SEM (*n* = 3 dishes per group; 30 oocytes per dish). Stage III oocytes (550–690 μm) were incubated at 28 °C in the dark for 260 min. (** *p* < 0.01).

Stage III oocytes from both 3–4 and 4–5 mpf mixed-sex co-housing females showed comparable meiotic competence, with no significant difference in DHP-induced GVBD rates (Figure 3B). However, the two age groups differed in morphometric dynamics. Post-incubation diameter increased in both age groups; this increase reached statistical significance only in the 4–5 mpf donors (*p* = 0.010), whereas the 3–4 mpf group showed a borderline trend (*p* = 0.056) (Figure 3A). Although mean Δdiameter (post-pre) did not differ significantly between ages (Figure 3C), the 4–5 mpf donors were more consistent at the dish level. Consequently, 4–5 mpf females were prioritized as donors for subsequent toxicology assays.

The 3–4 mpf window corresponds to early reproductive maturity in zebrafish, shortly after sexual maturity is established, whereas 4–5 mpf represents the period of stable reproductive function within the standard working range of ~3–6 months [29]. These two adjacent windows were selected to evaluate whether early-mature donors are equivalent to established-mature donors for IVM purposes, which is the most practically relevant comparison for workflow optimization within the routine donor age range. Extending the comparison below 3 mpf was not feasible, as females at our facility do not yet reliably yield sufficient stage III oocytes at earlier ages. Inclusion of fish older than 5–6 mpf was avoided because age-associated reproductive decline could introduce ovarian senescence as an additional factor [29], which is qualitatively distinct from the donor quality variation this study aimed to characterize. The comparable GVBD rates observed across the two age groups are consistent with the asynchronous nature of the zebrafish ovary [33], where stage III follicles coexist with multiple developmental stages regardless of donor age within the reproductive maturity window. That reproductive performance in this species reflects body condition and physiological state more than chronological age [30] may further explain the similar outcomes observed across the 3–5 mpf range examined here.

### 3.3. Husbandry Conditions Affect Oocyte Competence: Mixed-Sex Versus Separated

**Figure 4:**
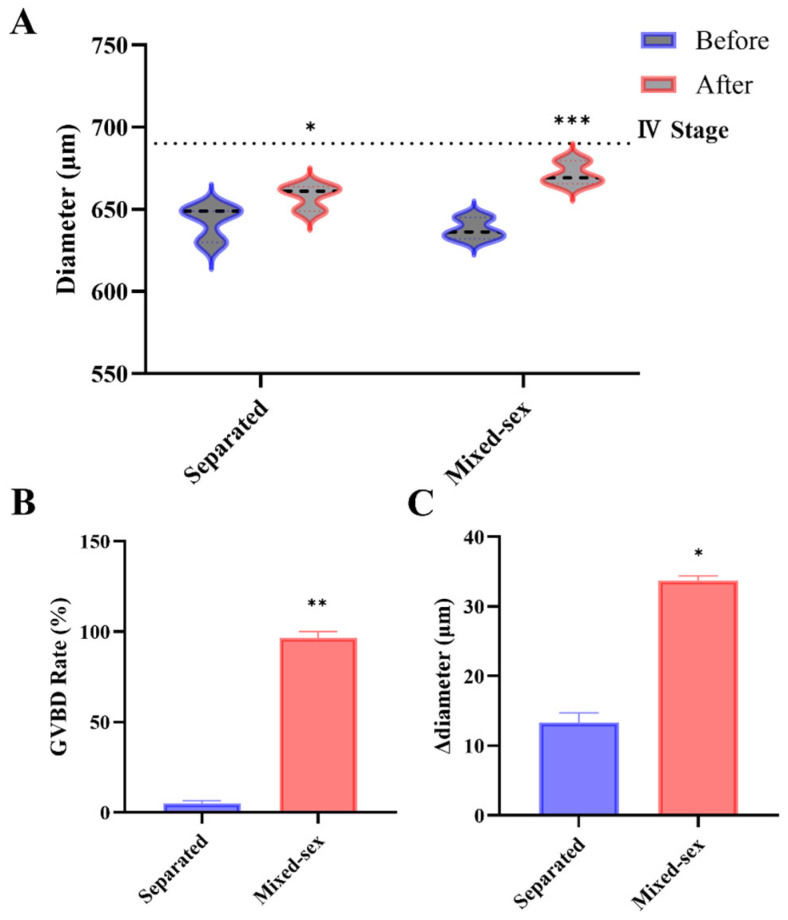
Husbandry condition (mixed-sex vs. separated) and IVM outcomes. (**A**) Oocyte diameter before and after incubation (paired comparisons within groups). (**B**) GVBD rate. (**C**) Δdiameter (post-pre) summarized as dish means. Data are mean ± SEM (*n* = 3 dishes per group; 30 oocytes per dish). Stage III oocytes were incubated at 28 °C in the dark for 260 min. (* *p* < 0.05; ** *p* < 0.01; *** *p* < 0.001).

Assessment of husbandry regimes indicated that the social environment influences donor quality. Stage III oocytes were collected from 4 to 5 mpf females maintained either in mixed-sex tanks or under separated housing and subjected to DHP-induced maturation. Under these conditions, GVBD was markedly higher in the mixed-sex group than in the separated group (dish means; *n* = 3 per group; *p* < 0.01) (Figure 4B). Oocyte diameter increased after incubation in both groups (paired comparisons within groups), with a larger Δdiameter observed in oocytes from mixed-sex donors; Δdiameter also differed between husbandry conditions (*p* < 0.05) (Figure 4A,C).

These results are consistent with prior reports that zebrafish reproductive physiology depends on social cues, particularly male-derived olfactory signals [34,35]. Zebrafish ovulation and spawning can depend on male-derived signals, and lack of male contact has been associated with increased follicular atresia and cystic ovary-like phenotypes, together with reduced reproductive output [9]. This comparison should nevertheless be interpreted cautiously. Ovaries from this entire batch appeared less developed at dissection than in other 4–5 mpf cohorts, resembling 3–4 mpf material in gross appearance; despite this, the mixed-sex group still produced acceptable GVBD, whereas the separated group showed an unusually low GVBD. The discrepancy indicates that husbandry conditions can substantially affect donor suitability for the IVM assay even when overall ovarian morphology is suboptimal. The batch-level morphological observation does not affect the selection of 4–5 mpf as the preferred donor window, which was based on the quantitative functional outcomes from the age-matched comparison in Section 3.2. While these data suggest that mixed-sex housing may be important for donor suitability, the magnitude of the effect should be regarded as preliminary and will require adequately powered, independently replicated trials for confirmation.

### 3.4. BTBPE Inhibits Oocyte Maturation with a Non-Monotonic-like Response Pattern

**Figure 5:**
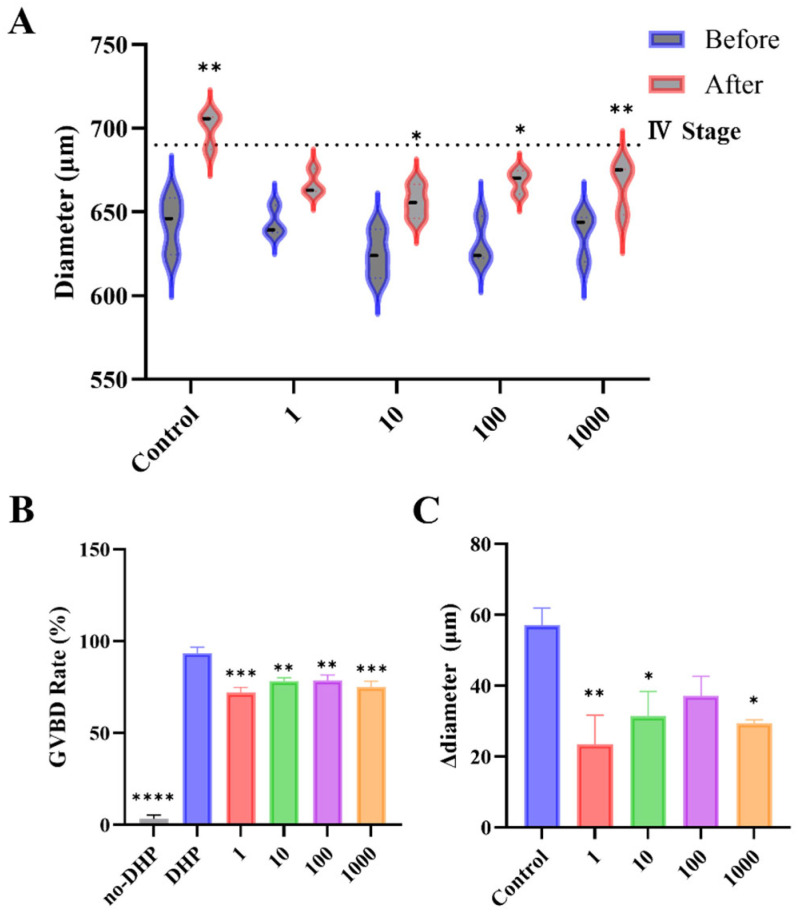
BTBPE suppresses DHP-induced zebrafish oocyte maturation with a non-monotonic-like response. (**A**) Oocyte diameter before and after incubation (paired comparisons within groups). (**B**) GVBD rate. (**C**) Δdiameter (post-pre) summarized as dish means. Data are mean ± SEM (*n* = 3 dishes per group; 30 oocytes per dish). Stage III oocytes (550–690 μm) were incubated at 28 °C in the dark for 260 min. (* *p* < 0.05; ** *p* < 0.01; *** *p* < 0.001, **** *p* < 0.0001).

To evaluate the direct effect of BTBPE on DHP-induced meiotic resumption, stage III oocytes isolated from 4 to 5 mpf mixed-sex co-housing females were matured in vitro in the presence of BTBPE (1–1000 nM). As expected, the hormone-free group (no-DHP) exhibited minimal spontaneous GVBD, whereas DHP robustly induced GVBD, confirming the responsiveness of the IVM model (Figure 5A). Relative to the DHP control, BTBPE significantly reduced GVBD at all tested concentrations (Fisher’s LSD; Figure 5B), indicating suppression of meiotic resumption under these conditions. Although inhibition was consistently observed, the magnitude of the response varied across concentrations and was not strictly dose-proportional, suggesting a non-monotonic-like concentration-response pattern.

Consistent with maturation-associated size dynamics, oocyte diameter increased after incubation in the DHP control (Figure 5A). BTBPE co-exposure altered these diameter dynamics, yielding smaller pre-post diameter shifts overall. When summarized as Δdiameter (post-pre) using dish means, Δdiameter was significantly lower than the DHP control at 1, 10, and 1000 nM, whereas the reduction at 100 nM was not statistically significant (pairwise comparisons vs. DHP; Figure 5C). The concordant reductions in GVBD and Δdiameter indicate that BTBPE inhibits DHP-induced maturation at the gamete level.

Placed in an exposure context, the lower end of the tested range is directly relevant to documented human internal exposure. Reported human serum concentrations of BTBPE reach approximately 0.33 nM in exposed populations [20], which is within the same order of magnitude as the lowest concentration tested here (1 nM). Tissue distribution data further indicate preferential partitioning into lipid-rich compartments, including the gonads, so local follicular concentrations after in vivo bioaccumulation may exceed serum-equivalent values. Accordingly, the higher concentrations (10–1000 nM) also serve to approximate the elevated tissue concentration expected in lipid-rich ovaries following in vivo bioaccumulation. Consequently, the suppression observed at 1 nM indicates that BTBPE may interfere with oocyte maturation at concentrations relevant to documented human exposure.

The non-monotonic-like pattern observed in our GVBD assay (Figure 5B) is a recognized feature of many EDCs. Such patterns can arise when a compound binds multiple receptor targets, or when receptor-mediated effects at low concentrations give way to other mechanisms at higher concentrations [36]. The BTBPE pattern observed here, consistent with GVBD suppression without strict dose proportionality, fits this framework, although the specific receptor interactions remain unresolved.

At the phenotypic level, previous zebrafish IVM studies offer context for the BTBPE effects reported here. Chemical effects on meiotic resumption vary considerably across compounds in this model: some agents suppress hormone-induced GVBD, while others accelerate or promote maturation. Within a single screening study, endosulfan inhibited induced GVBD at concentrations as low as 0.03 µM, while iprodione promoted rather than inhibited maturation [16]. Among brominated flame retardants, dietary TBCO exposure reduced in vitro oocyte maturation competence [17], a directional outcome consistent with the BTBPE-induced GVBD suppression observed here. Taken together, these results raise the possibility that impaired meiotic competence may be a shared concern across brominated flame retardants rather than an isolated response to individual compounds.

In a related context, another study on BPA demonstrated that it inhibits the spontaneous in vitro maturation of defolliculated oocytes at concentrations of 10–100 nM by acting at Gper to elevate cAMP and maintain meiotic arrest [15]. However, the experimental context differs from the present study in a relevant way, as DHP promotes meiotic resumption by binding mPRα and suppressing cAMP via Gi signaling [31,37], acting in a direction opposite to the estrogen-mediated arrest that BPA reinforces. BTBPE was tested here under DHP induction rather than as a modulator of spontaneous maturation, and the non-monotonic-like inhibitory pattern it produced across a 1000-fold concentration range contrasts with the graded BPA response [15]. These differences in both experimental context and concentration-response profile suggest that BTBPE and BPA interfere with oocyte maturation through distinct mechanisms. That structurally diverse EDCs can converge on suppression of DHP-induced meiotic resumption while acting through different upstream pathways is itself a toxicologically relevant observation, and gamete-level assays are well positioned to capture such distinctions.

By removing HPG axis regulation and avoiding whole-animal endocrine confounding, the zebrafish IVM model is uniquely suited to isolate these chemical effects at the gamete level [16,25]. Building upon this inherent advantage of the model, the workflow refinements reported here target two practical sources of variation in existing protocols. Donor-condition optimization provides a documented basis for female selection, a factor that affects batch quality but has received limited systematic attention in the IVM-based reproductive toxicology literature. Real-time diameter quantification replaces subjective visual staging with a continuous, recorded metric that can be audited and reproduced across laboratories. Reporting Δdiameter alongside GVBD also raises the informational yield per assay, as concordant suppression of both endpoints across all BTBPE concentrations provides stronger evidence of impaired maturation competence than either endpoint considered alone. Together, these refinements make the zebrafish IVM assay a practical screen for the reproductive toxicity of emerging brominated contaminants.

## 4. Conclusions

This study established a refined zebrafish oocyte IVM workflow that integrates GVBD assessment with real-time, image-based oocyte diameter quantification and QC reporting. The workflow operated reliably, with most oocytes detected automatically and only limited manual intervention required. Donor optimization indicated broadly comparable GVBD outcomes between 3–4 and 4–5 mpf females, whereas 4–5 mpf donors showed more consistent diameter dynamics at the dish level and were therefore selected for subsequent assays. Oocytes from females maintained under separated housing also showed markedly reduced maturation performance compared with mixed-sex co-housing, indicating that husbandry conditions contribute to donor suitability for IVM, although this effect should be regarded as preliminary. Under DHP induction, BTBPE (1–1000 nM) consistently suppressed GVBD and reduced Δdiameter relative to DHP controls at most concentrations tested, with a non-monotonic-like concentration-response pattern. The concordant changes in both endpoints indicate that BTBPE impairs zebrafish oocyte maturation competence under defined in vitro conditions. Mechanistic dissection and whole-animal validation are needed to determine whether this gamete-level effect translates into in vivo reproductive impairment.

## Data Availability

The original contributions presented in this study are included in the article. Further inquiries can be directed to the corresponding authors.

## Abbreviations

The following abbreviations are used in this manuscript:

BTBPE	1,2-Bis(2,4,6-tribromophenoxy)ethane
DHP	17α,20β-dihydroxy-4-pregnen-3-one
IVM	In Vitro Maturation
GVBD	Germinal Vesicle Breakdown
